# DNA-methylation-mediated silencing of miR-486-5p promotes colorectal cancer proliferation and migration through activation of PLAGL2/IGF2/β-catenin signal pathways

**DOI:** 10.1038/s41419-018-1105-9

**Published:** 2018-10-10

**Authors:** Xiangxiang Liu, Xiaoxiang Chen, Kaixuan Zeng, Mu Xu, Bangshun He, Yuqin Pan, Huiling Sun, Bei Pan, Xueni Xu, Tao Xu, Xiuxiu Hu, Shukui Wang

**Affiliations:** 1 General Clinical Research Center, Nanjing First Hospital, Nanjing Medical University, 210006 Nanjing, China; 2 School of Medicine, Southeast University, 210009 Nanjing, China

## Abstract

As one of the most common cancers worldwide, colorectal cancer (CRC) causes a large number of mortality annually. Aberrant expression of microRNAs (miRNAs) is significantly associated with the initiation and development of CRC. Further investigations regarding the regulatory mechanism of miRNAs is warranted. In this study, we discovered that miR-486-5p was remarkably downregulated in CRC, which partially results from higher DNA methylation in the promoter region detected by using methylation-specific PCR, bisulfite sequencing PCR, and DNA demethylation treatment. Besides, decreased miR-486-5p was obviously associated with advanced TNM stage, larger tumor size, lymphatic metastasis, and poor prognosis in CRC. Upregulated miR-486-5p inhibited the proliferation and migration of CRC through targeting PLAGL2 expression and subsequent repressing IGF/β-catenin signal pathways both in vitro and in vivo. Notably, plasma miR-486-5p expression was significantly upregulated in CRC patients and we identified plasma miR-486-5p as a novel diagnostic biomarker of CRC using receiver operating characteristic (ROC) curve analysis. Moreover, exploration in GEO dataset revealed that circulating miR-486-5p is tumor derived through being packaged into secretory exosomes. Taken together, our data demonstrated that miR-486-5p promotes colorectal cancer proliferation and migration through activation of PLAGL2/IGF2/β-catenin signal pathway, which is a promising therapeutic target of CRC treatment.

## Introduction

Colorectal cancer (CRC) is one of the most common malignancies worldwide, causing almost 700,000 deaths annually^1^. According to the recent report of The International Agency for Research on Cancer, CRC is responsible for 6.3% of all tumorous mortality in Chinese population^2^. Because of the absence of specific and sensitive biomarkers, patients with CRC are usually diagnosed at an advanced stage and consequently suffer from poor 5-year survival rate^3^. In spite of improvements of surgical resection and adjuvant chemotherapies, the prognosis of CRC patients remains unfavorable due to the local and distant metastases^4,5^. Mounting studies have demonstrated that the pathogenesis of CRC results from the mutations of oncogenes and tumor-suppressor genes^6,7^. In order to identify sensitive and specific biomarkers and effective therapeutic strategies of CRC, it is important to improve the understanding of the molecular mechanisms underlying CRC progression.

A growing body of evidence has demonstrated that microRNAs (miRNAs), a family of endogenous small non-coding RNAs (average 22 nucleotides in length), can functionally carry out biological effects through direct binding to the 3′ untranslated regions (UTRs) of targets inducing mRNA degradation and/or translational repression^8^. Emerging studies reported that miRNAs regulate approximate 60% protein-coding genes that are linked with several biological processes, including cell proliferation, apoptosis, invasion, chemosensitivity, and so on^9–11^. Recently, increasing evidence demonstrated that miR-486-5p acts as a tumor inducer or suppressor in multiple human malignancies^12–14^. Besides, The Cancer Genome Atlas (TCGA) database illustrated that miR-486-5p is one of the most downregulated miRNAs in CRC. However, the regulatory mechanisms of miR-486-5p in CRC progression remains elusive.

Pleomorphic adenoma gene-like 2 (PLAGL2), a member of the pleomorphic adenoma gene (PLAG)-family, is a zinc-finger transcription factor that is located in the nucleus^15,16^. PLAGL2 was initially found in mouse cell lines^17^. Whereafter, accumulating studies discovered that PLAGL2 is overexpressed in multiple cancers and plays a critical role in cancer progression^18,19^. However, the crosstalk between PLAGL2 and miRNAs remains under further exploration.

In this study, we demonstrated that miR-486-5p was significantly downregulated in CRC mediated by the DNA methylation of its promoter. Overexpression of miR-486-5p inhibited CRC cell proliferation and invasion both in vitro and in vivo. The regulatory effects of miR-486-5p in CRC are associated with the amplification of PLAGL2 and corresponding downstream insulin-like growth factor-2 (IGF2) and β-catenin signals. Furthermore, we discovered that increased plasma miR-486-5p serves as a novel promising diagnostic biomarker to discriminate between CRC patients and healthy individuals with non-invasion.

## Results

### MiR-486-5p expression is downregulated in CRC tissues and cell lines

**Fig. 1:**
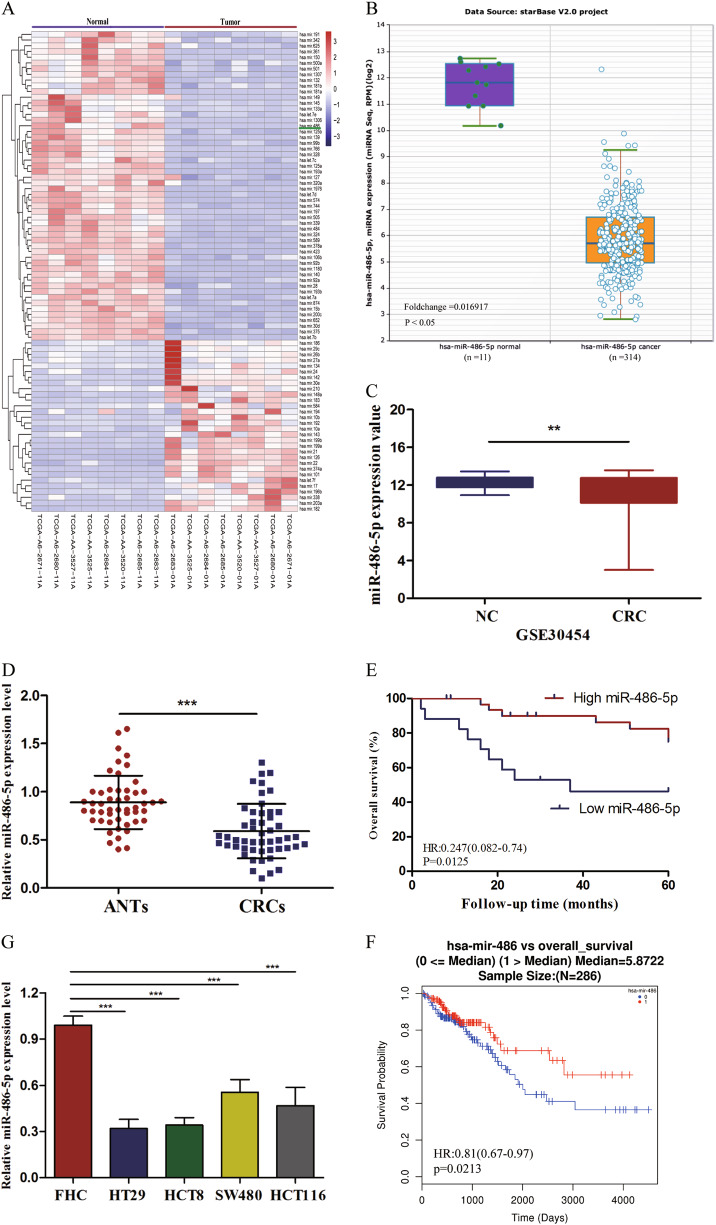
MiR-486-5p is remarkably downregulated in CRC and predicts poor prognosis. **a**–**c** TCGA, Starbase 2.0, and GEO databases exhibited miR-486-5p is significantly downregulated in CRC. **d** miR-486-5p is significantly downregulated in CRC compared to ANTs. **e**, **f** Our study and TCGA database revealed that CRC patients with low miR-486-5p expression had lower overall survival rates than patients with high miR-486-5p expression. Data are represented as means ± S.D. from at least three independent experiments. **g** miR-486-5p is significantly downregulated in CRC cells compared to FHC. *p < 0.05, **p < 0.01, ***p < 0.001

TCGA database illustrated that miR-486-5p is one of the most downregulated miRNAs in CRC (Fig. 1a) supported by Starbase v2.0 database and GEO database (GSE30454), which exhibited a consistent result (Fig. 1b, c). In our study, we analyzed the expression of miR-486-5p in 50 paired CRC tissues and adjacent normal tissues (ANTs) by quantitative real-time polymerase chain reaction (qRT-PCR). The result validated that miR-486-5p expression was remarkably reduced in CRC tissues compared to ANTs (Fig. 1d). Soon afterwards, we divided 50 CRC patients into high miR-486-5p group (value >0.508, *n* = 33) and low miR-486-5p group (value ≤0.508, *n* = 17) according to the median value of relative miR-486-5p expression level and analyzed the association between miR-486-5p expression and clinicopathological characteristics of patients (Table 1). Chi-square test showed that low miR-486-5p expression was significantly correlated with advanced tumor, node, metastasis (TNM) stage (*p* = 0.001), lymphatic metastasis (*p* = 0.016), and larger tumor size (*p* = 0.013). We next performed univariate and multivariate logistic regression models to analyze the correlation of miR-486-5p expression and overall survival (OS) of CRC patients. Univariate analysis exhibited that miR-486-5p expression (hazard ratio [HR] = 0.306, 95% confidence interval [CI] 0.11–0.83, *p* = 0.019), TNM stage (HR = 4.44, 95% CI 1.42–13.89, *p* = 0.01), histology grade (HR = 4.67,95% CI 1.06–20.4, *p* = 0.042), distant metastasis (HR = 5.92, 95% CI 1.99–17.54, *p* = 0.001), and lymphatic metastasis (HR = 2.81, 95% CI 1.04–7.58, *p* = 0.041) were significantly correlated with OS of CRC patients (all *p* < 0.05). Multivariate analysis indicated that TNM stage (HR = 3.87, 95% CI 1.13–13.33, *p* = 0.031) and histology grade (HR = 5.35, 95% CI 1.09–26.36, *p* = 0.038) were independent prognostic factors of CRC patients (Table 2). Kaplan–Meier survival analysis proved that patients with higher miR-486-5p expression had better 5-year survival rate than those with lower miR-486-5p expression (Fig. 1e), which was consistent with the result of TCGA database (Fig. 1f). Additionally, the expression of miR-486-5p in CRC cell lines (HT29, HCT8, HCT116, SW480) exhibited differently lower levels than that in FHC (Fig. 1g).

**Table 1:** Association of miR-486-5p expression with clinicopathological parameters of CRC patients

Variables	miR-486-5p expression		*p* Value
	High	Low	
*Gender*			
Male	20	9	
Female	13	8	0.603
*Age, years*			
>63	17	6	
≤63	16	11	0.267
*Tumor location*			
Colon	19	10	
Rectum	14	7	0.933
*TNM stage*			
I+II	22	3	
III+IV	11	14	0.001
*Histologic grade*			
1+2	13	5	
3	20	12	0.486
*Lymphatic metastasis*			
N0	25	7	
N1+N2	8	10	0.016
*Distant metastasis*			
M0	30	13	
M1	3	4	0.21
*Tumor size*			
≤4 cm	31	11	
>4 cm	2	6	0.013

**Table 2:** Univariate and multivariate analysis for OS of CRC patients

Variables	Univariate analysis			Multivariate analysis		
	HR	95% CI	*p* Value	HR	95% CI	*p* Value
Gender (male, female)	2.599	0.838–8.066	0.089			
Age (>63, ≤63), years	0.762	0.277–2.098	0.599			
Tumor location (colon, rectum)	0.598	0.224–1.598	0.306			
TNM stage (III+IV, I+II)	4.44	1.42–13.89	0.01	3.87	1.13–13.33	0.031
Histologic grade (3, 1+2)	4.67	1.06–20.4	0.042	5.35	1.09–26.32	0.038
Lymphatic metastasis (N1+N2, N0)	2.81	1.04–7.58	0.041	1.93	0.65–5.68	0.235
Distant metastasis (M1, M0)	5.92	1.99–17.54	0.001	2.78	0.76–10.1	0.121
Tumor size (>4 cm, ≤4 cm)	4.062	0.92–17.94	0.064	4.51	0.89–22.67	0.067
miR-486-5p expression (high, low)	0.306	0.11–0.83	0.019	0.68	0.21–2.22	0.523

### MiR-486-5p expression is regulated by DNA methylation of its promoter

**Fig. 2:**
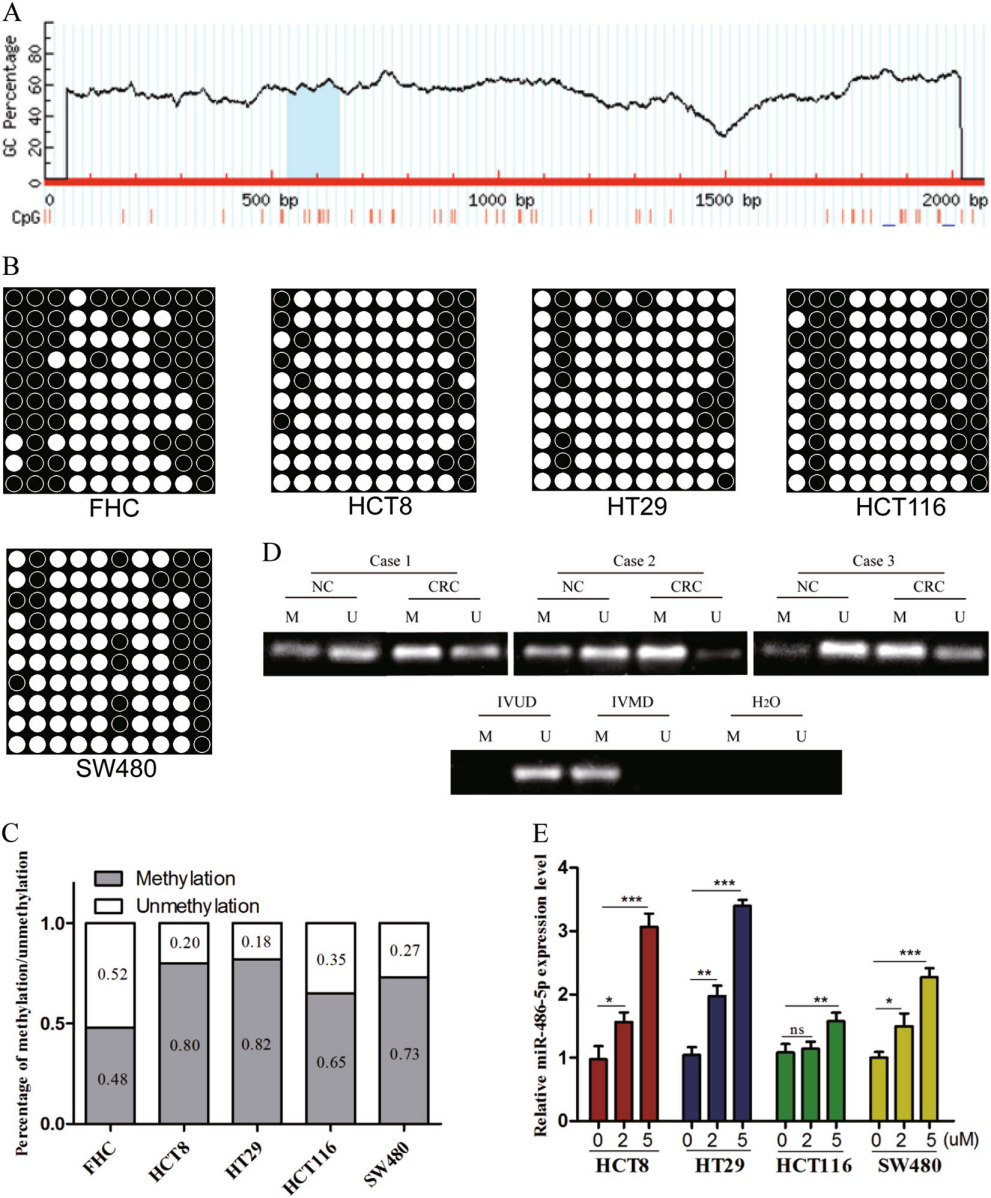
MiR-486-5p expression is regulated by DNA methylation of its promoter. **a** MethPrimer website predicted that a CpG island is located at the promoter region of miR-486-5p. **b**, **c** BSP analysis demonstrated that methylated CG sites in CRC cell lines were more than that in FHC. **d** MSP analysis uncovered hypermethylation of the miR-486-5p promoter in CRC tissues when compared to ANTs. **e** The expression of miR-486-5p restored in CRC cell lines using 5-aza-2′-deoxycytidine. Data are represented as means ± S.D. from at least three independent experiments. *p < 0.05, **p < 0.01, ***p < 0.001

To investigate the upstream mechanism involved in miR-486-5p downregulation in CRC, we predicted a CpG island in the promoter region of miR-486-5p using methylation software analysis (http://www.urogene.org/methprimer/) (Fig. 2a). Bisulfite sequencing PCR (BSP) analysis demonstrated that methylated CG sites in CRC cell lines were more than that in FHC (Fig. 2b, c). In addition, using methylation-specific PCR (MSP), we frequently observed hypermethylation of the miR-486-5p promoter in CRC tissues when compared to ANTs (Fig. 2d). After exposure to 5-aza-2′-deoxycytidine, a kind of DNA demethylating agent, the expression of miR-486-5p was restored in CRC cell lines (Fig. 2e).

### MiR-486-5p suppresses CRC cell proliferation and migration in vitro and in vivo

**Fig. 3:**
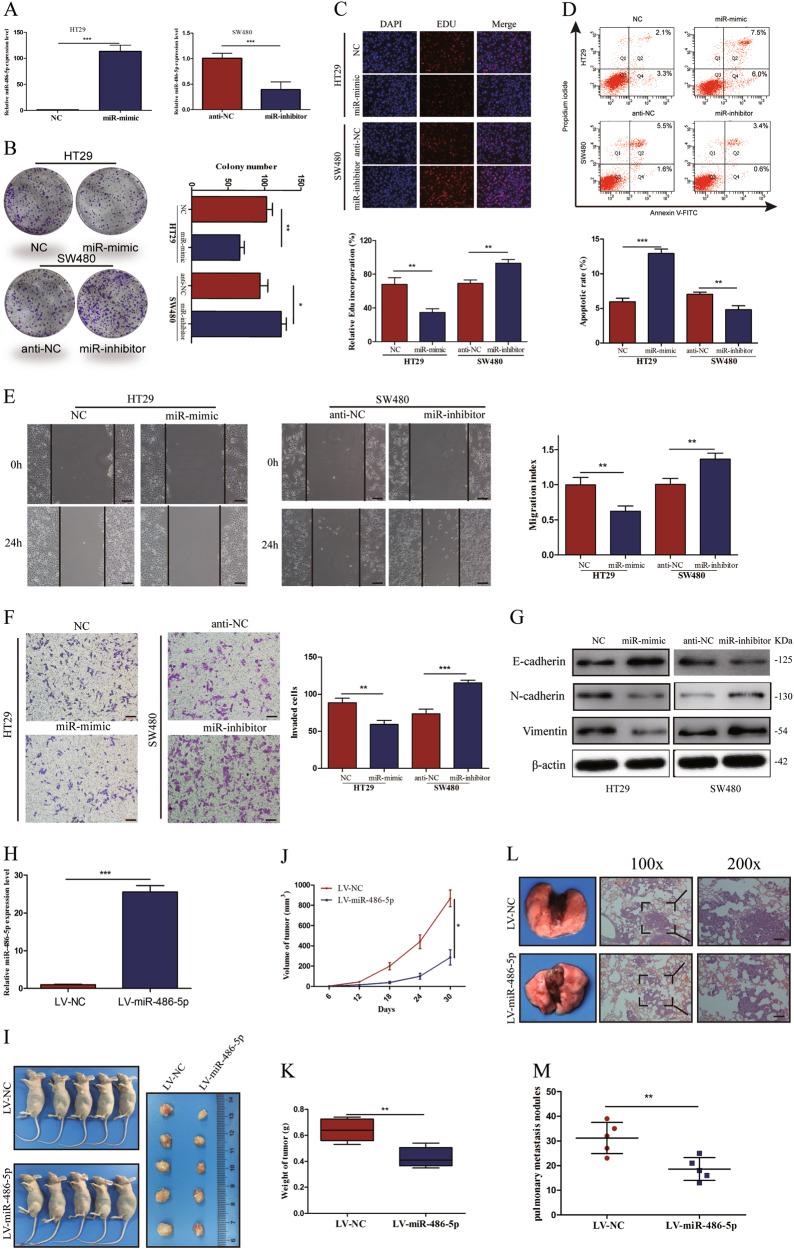
MiR-486-5p suppresses CRC cell proliferation and migration in vitro and in vivo. **a** MiR-486-5p expression was significantly upregulated after transfection with miR-mimic but downregulated after transfection with miR-inhibitor. **b**, **c** Colony-formation and EdU assays revealed that miR-486-5p suppresses CRC cell proliferation. **d** Flow cytometric apoptosis analysis of HT29 cells and SW480 cells transfected with miR-mimic or miR-inhibitor. **e**, **f** Wound healing assay and transwell assay uncovered that miR-486-5p repress CRC cells' migration and invasion abilities. **g** Western blot assay exhibited that upregulated miR-486-5p increased the expression of E-cadherin and inhibited the expression of N-cadherin and Vimentin. **h** MiR-486-5p is stably overexpressed using a lentivirus-based transfection system. **i**, **j** The tumors grew more slowly in the LV-miR-486-5p group when compared to the LV-NC group. **k** The tumor weights in the LV-miR-486-5p group were lower than those in the LV-miR-NC group. **l**, **m** Fewer lung metastatic nodules were searched in the LV-miR-486-5p group compared to that in the LV-miR-NC group. Data are represented as means ± S.D. from at least three independent experiments. Scare bar = 50 μm. *p < 0.05, **p < 0.01, ***p < 0.001

In order to identify and validate the effect of miR-486-5p on cellular behavior, HT29 cells were transfected with miRNA mimics (miR-mimic) and SW480 cells were transfected with miRNA inhibitor (miR-inhibitor), and qRT-PCR was used to detect the transfection efficiency (Fig. 3a). The colony-formation assay and 5-ethynyl-20-deoxyuridine (EdU) assay exhibited that miR-486-5p overexpression significantly suppressed cell proliferation in HT29 cells, whereas miR-486-5p downregulation enhanced the proliferative activity of SW480 cells (Fig. 3b, c). Annexin V staining revealed that the percentage of cells undergoing apoptosis increased in HT29 cells and decreased in SW480 cells after transfection with miR-mimic and miR-inhibitor, respectively (Fig. 3d). Wound healing and transwell assays revealed that miR-mimic-treated HT29 cells underwent a significant reduction in motility activities, whereas the inhibition of endogenous miR-486-5p increased the motility potential of SW480 cells (Fig. 3e, f). Western blot (WB) assay exhibited that upregulated miR-486-5p increased the expression of epithelial markers (E-cadherin) and inhibited the expression of mesenchymal markers (N-cadherin, Vimentin) (Fig. 3g).

To investigate the biological function of miR-486-5p in CRC cells' proliferation and migration in vivo, we established HT29 cells with miR-486-5p stable overexpression using a lentivirus-based system (LV-miR-486-5p and LV-NC). qRT-PCR analysis confirmed that the expression of miR-486-5p in LV-miR-486-5p-transfected HT29 cells remarkably increased compared to that in LV-NC-transfected HT29 cells (Fig. 3h). Then HT29 cells with stable transfection were subcutaneously implanted into nude mice. After implantation, as expected, the tumors grew more slowly in the LV-miR-486-5p group when compared to the LV-NC group (Fig. 3i, j). Besides, the weights of tumors in LV-miR-486-5p group were clearly lower than that in the LV-miR-NC group (Fig. 3k).

To exam whether increased miR-486-5p influences the metastasis activity of CRC cells in vivo, the immunodeficient mice were injected with HT29 cells transfected with LV-miR-486-5p or LV-NC through the tail vein. Seven weeks after the injection, these mice were sacrificed and the number of tumor nodules in the lungs were observed. When compared to that in LV-miR-NC group, fewer tumorous nodules were searched in the lungs of mice in LV-miR-486-5p group (Fig. 3l, m).

### MiR-486-5p targets PLAGL2 directly

**Fig. 4:**
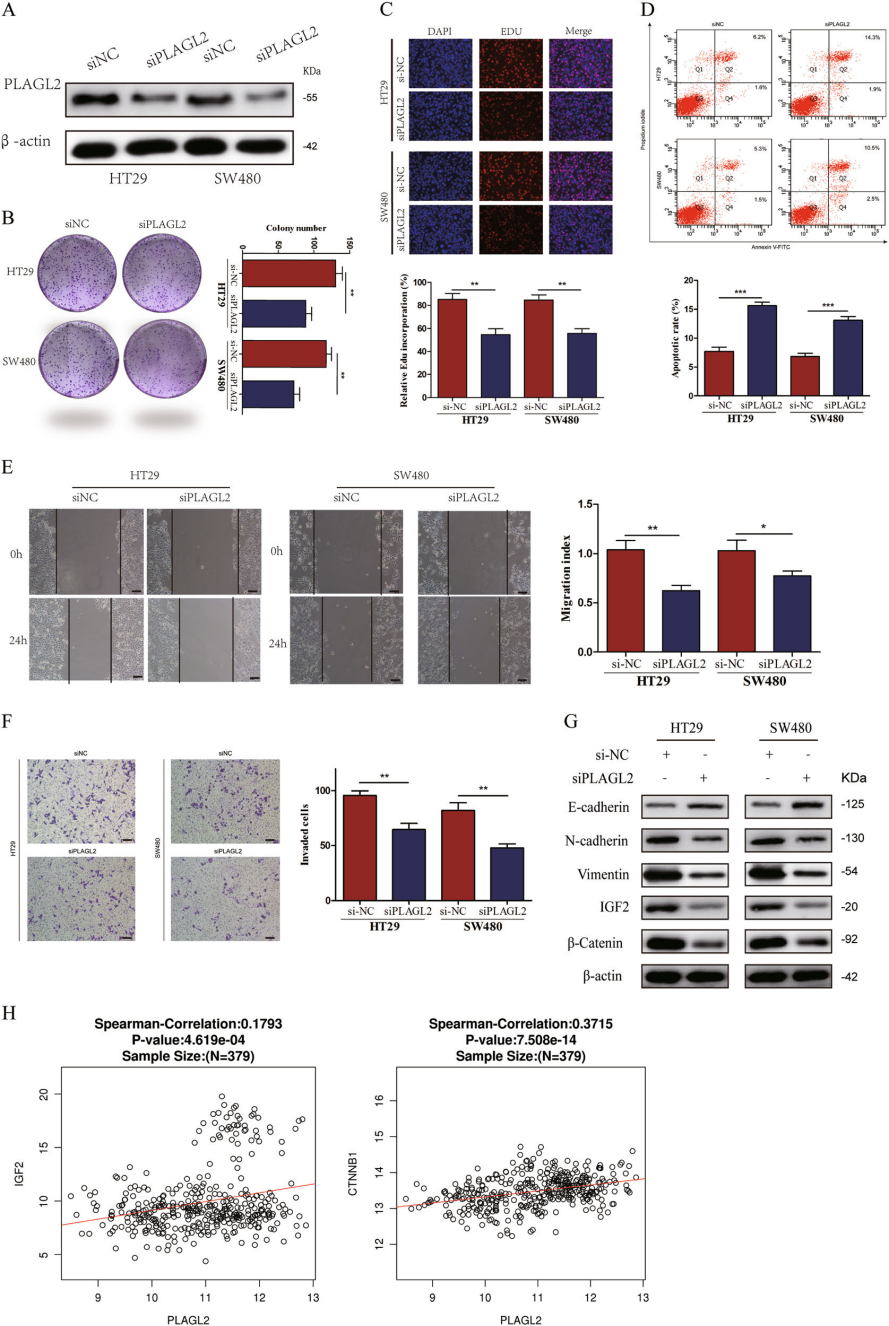
Inhibition of PLAGL2 attenuates CRC cells' proliferation and migration. **a** Western blot result of PLAGL2 protein after cells transfected with siPLAGL2. **b**, **c** Colony-formation assay and EdU assay revealed that transfection with siPLAGL2 suppresses CRC cell proliferation. **d** Flow cytometric apoptosis analysis of HT29 cells and SW480 cells transfected with siPLAGL2. **e**, **f** Wound healing assay and transwell assay uncovered that inhibition of PLAGL2 represses CRC cells' migration and invasion abilities. **g** Western blot assay exhibited that siPLAGL2 increased the expression of E-cadherin and inhibited the expression of N-cadherin, Vimentin, β-catenin, and IGF2. **h** IGF2 and β-catenin expression was positively correlated with PLAGL2 expression in TCGA database. Data are represented as means ± S.D. from at least three independent experiments. Scare bar = 50 μm. *p < 0.05, **p < 0.01, ***p < 0.001

To further investigate the molecular mechanisms of miR-486-5p-modulated suppression of CRC proliferation and migration, four different public bioinformatics algorithms (TargetScan, miRDB, Tarbase, and miRanda) and TCGA database were used. After integrated analysis of results from four bioinformatics algorithms, five potential targets of miR-486-5p were found (H3F3B, ATXN7L3, PLAGL2, ARID4B, and BTAF1) (Fig. 4a). However, only 2 target genes (PLAGL2 and ARID4B) expression were proved to be significantly correlated with the miR-486 expression by analysis of 295 specimens in TCGA database (Fig. 4b). qRT-PCR and WB analysis showed that the expression of PLAGL2 dramatically changed in the miR-mimic and miR-inhibitor groups compared with that in NC and anti-NC groups, respectively (Fig. 4c, d). Additionally, in vivo, the expression of PLAGL2 protein in LV-miR-486-5p-transfected subcutaneous tumors was remarkably downregulated compared to that in LV-miR-NC-transfected subcutaneous tumors (Fig. 6i). Next, we identified a putative binding site of miR-486-5p with highly conserved complementarity in the 3′-UTR of PLAGL2 using a computational prediction (Fig. 4e). Dual-luciferase reporter assay demonstrated that the miR-486-5p overexpression significantly decreased the firefly luciferase reporter activity of the PLAGL2 wild-type (wt) vector but not the mutant (mut) vector in 293T cells (Fig. 4f).

### The expression of PLAGL2 is upregulated in CRC tissues and cell lines

To assess the expression of PLAGL2 mRNA and protein in cells, qRT-PCR and WB analysis was conducted. The results revealed that the expression levels of PLAGL2 were significantly higher in CRC cell lines when compared to FHC (Fig. 4g, i). In addition, qRT-PCR and immunohistochemical (IHC) analysis were done to further evaluate the expression of PLAGL2 mRNA and protein in 50 matched CRC tissues and ANTs. The results from qRT-PCR showed a significant upregulation of PLAGL2 mRNA, which is consistent with the results of TCGA database (Fig. 4h, k) and a negative correlation was found between PLAGL2 mRNA and miR-486-5p expression in CRC tissues (Fig. 4j). IHC analysis revealed that protein levels of PLAGL2 were highly expressed in CRC tissues compared to ANTs (Fig. 4l). Clinical data analysis from TCGA database and our study discovered that PLAGL2 expression has no relationship with patients’ OS.

### Inhibition of PLAGL2 attenuates CRC cells' proliferation and migration

**Fig. 5:**
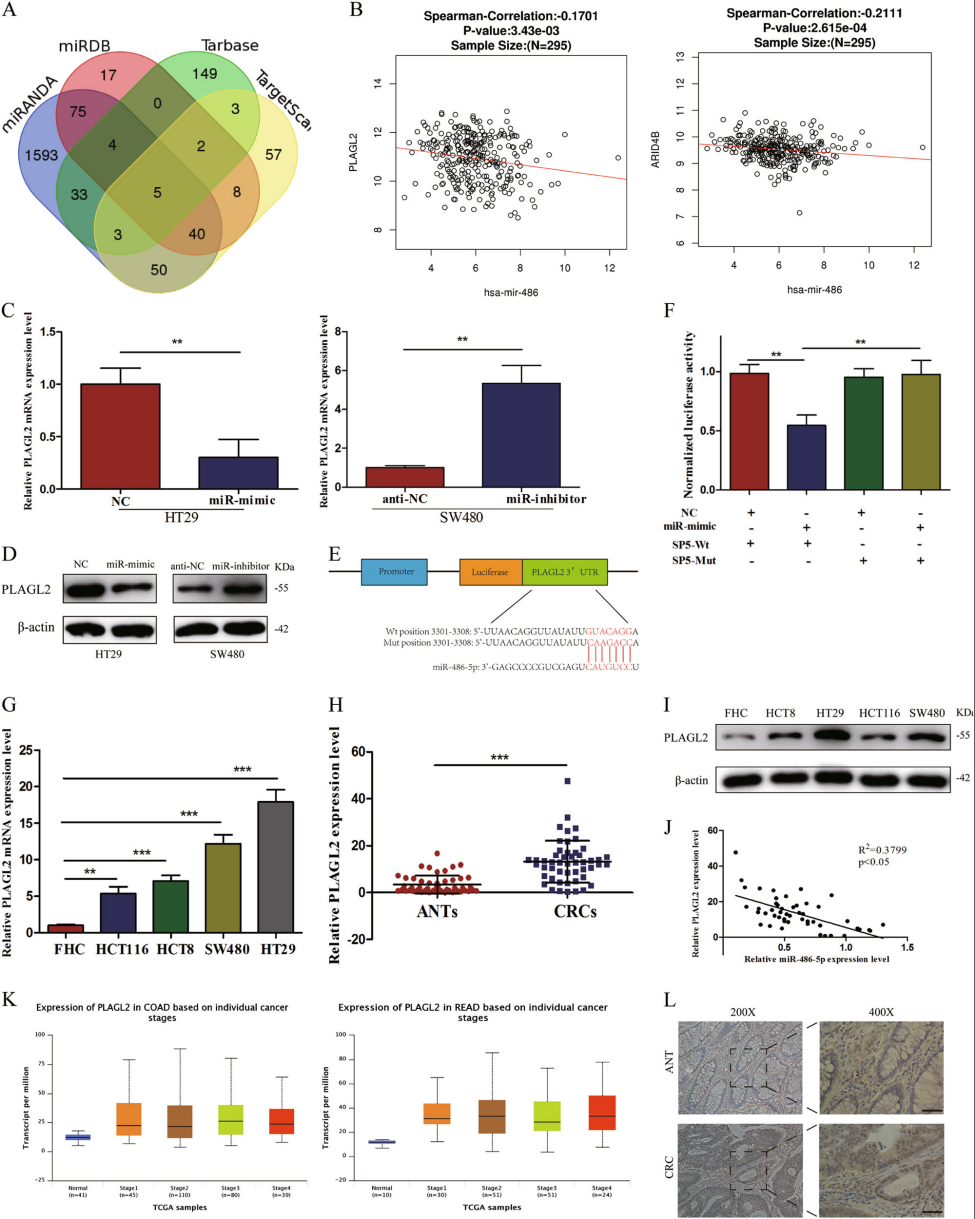
PLAGL2 PLAGL2 is a direct target of miR-486-5p and upregulated in CRC tissues and cell lines. **a** Five potential targets of miR-486-5p selected by public bioinformatics algorithms. **b** PLAGL2 and ARID4B expression were negatively correlated with miR-486 expression in TCGA database. **c**, **d** qRT-PCR and WB analysis showed that miR-486-5p could inhibit PLAGL2 expression both in mRNA and protein levels. **e** Predicted binding site in the 3′-UTRs of PLAGL2. **f** Dual luciferase reporter assays in 293T cells. **g**, **i** The expression levels of both PLAGL2 mRNA and protein were significantly higher in CRC cell lines. **h**, **j**, **l** qRT-PCR and IHC analysis demonstrated that PLAGL2 was significantly higher in CRC tissues and negatively correlated with miR-486-5p expression. **k** TCGA database demonstrated that PLAGL2 was significantly higher in CRC tissues. Data are represented as means ± S.D. from at least three independent experiments. Scare bar = 50 μm. *p < 0.05, **p < 0.01, ***p < 0.001

Previous studies have reported that transcription factor PLAGL2 was increased in a variety of cancers and promoted cancer cells' proliferation and metastasis^20–22^. To further determine the functional role of PLAGL2 in miR-486-5p-induced inhibition of proliferation and migration in CRC, the expression of PLAGL2 was knocked down using siPLAGL2 and WB assay confirmed that siPLAGL2 specifically decreased the PLAGL2 protein expression level in HT29 and SW480 cells (Fig. 5a). Colony-formation and EdU assays showed that siPLAGL2 significantly suppresses cells' proliferation (Fig. 5b, c). Annexin V staining revealed that the percentage of cells undergoing apoptosis increased after exposure to siPLAGL2 compared to si-NC (Fig. 5d). Wound healing and transwell assays exhibited that cells transfected with siPLAGL2 underwent a significant reduction in motility activities (Fig. 5e, f). WB assay exhibited that siPLAGL2 increased the expression of E-cadherin and inhibited the expression of N-cadherin and Vimentin (Fig. 5g).

Given the essential role of PLAGL2 in modulating Wnt/β-catenin and IGF2 signals^19,23,24^, we detected the effect of siPLAGL2 on both Wnt/β-catenin and IGF2 signal pathways in HT29 and SW480 cells via WB analysis to detect the changes of β-catenin and IGF2 expression. SiPLAGL2 significantly decreased β-catenin and IGF2 protein expression levels, which is similar to the result of miR-486-5p overexpression toward β-catenin and IGF2 expression (Fig. 5g). In addition, IGF2 and β-catenin expression was positively correlated with PLAGL2 expression in TCGA database (Fig. 5h).

### Downregulated PLAGL2 effectively reverses miR-inhibitor-induced progression of CRC cells

**Fig. 6:**
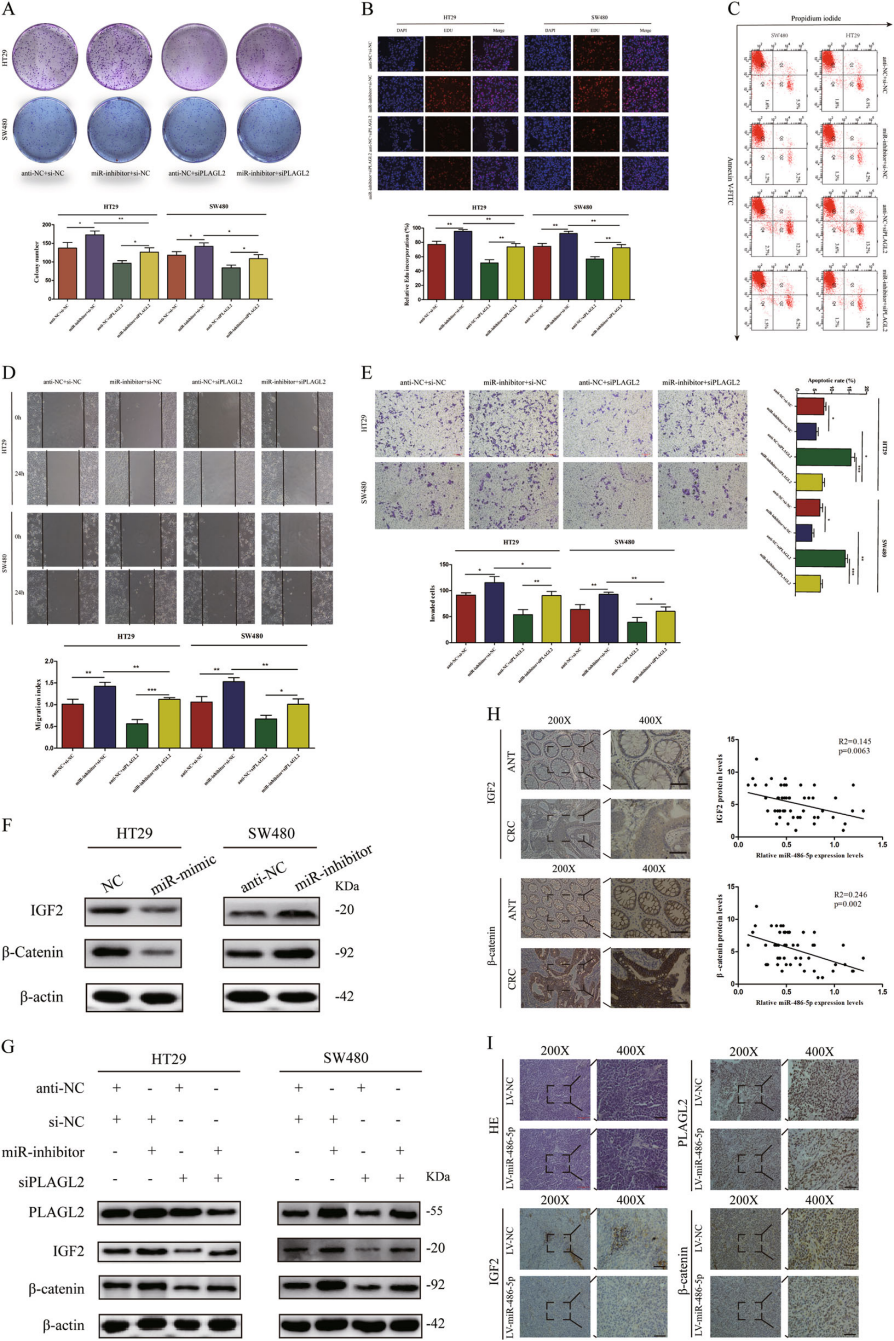
MiR-486-5p modulates CRC cells proliferation via PLAGL2/β-catenin /IGF2 signaling pathways. Downregulated PLAGL2 effectively reverses miR-inhibitor-induced **a**, **b** proliferation, **c** apoptosis, **d** migration, and **e** invasion ability of CRC cells. **f** Western blot analysis revealed that miR-486-5p inhibits the expression of IGF2 and β-catenin. **g** The inhibit effects of miR-486-5p on IGF2 and β-catenin were abolished by transfection of siPLAGL2. **h** The protein levels of IGF2 and β-catenin in CRC were significantly higher than that in adjacent normal tissues and negatively correlated with the miR-486-5p expression. **i** Representative PLAGL2, IGF2, and β-catenin staining in Xenograft subcutaneous tissues. Scare bar = 50 μm. *p < 0.05, **p < 0.01, ***p < 0.001

Subsequently, we wondered whether miR-486-5p regulated CRC cells' progression through direct inhibition of PLAGL2. HT29 and SW480 cells were co-transfected with miR-486-5p inhibitor and si-PLAGL2 vectors. The colony-formation assay showed that CRC cells co-transfected with miR-inhibitor and si-PLAGL2 exhibited fewer clones compared to the cells transfected with miR-inhibitor alone (Fig. 6a). Besides, EdU assay indicated downregulated miR-486-5p along with si-PLAGL2 inhibited CRC cells' proliferation when compared to downregulated miR-486-5p alone (Fig. 6b). Moreover, more apoptotic cells were exhibited in the miR-inhibitor+si-PLAGL2 group as compared to the miR-inhibitor+si-NC group (Fig. 6c). Wound healing and transwell assays revealed that miR-486-5p inhibitor together with si-PLAGL2 significantly weakened HT29 and SW480 cells' migration (Fig. 6d) and invasion (Fig. 6e) ability compared with the miR-486-5p inhibitor alone.

### MiR-486-5p modulates CRC cells' proliferation via PLAGL2/β-catenin/IGF2 signaling pathways

Data described above exhibited that miR-486-5p directly targeted PLAGL2 and siPLAGL2 inhibited cell proliferation and migration through downregulation of β-catenin and IGF2 in CRC. Subsequently, we investigated whether the β-catenin and IGF2 expression was involved in miR-486-5p-PLAGL2-regulated cell proliferation and migration. WB analysis showed that miR-mimic inhibited PLAGL2/β-catenin/IGF2 signaling pathways in HT29 cells, whereas miR-inhibitor enhanced PLAGL2/β-catenin/IGF2 signals in SW480 cells (Fig. 6f). Furthermore, anti-miR-486-5p-induced β-catenin and IGF2 expression was partially reversed by siPLAGL2 in both HT29 and SW480 cell lines (Fig. 6g).

In order to investigate the correlation between β-catenin, IGF2, and miR-486-5p in clinical specimens, we conducted IHC analysis in CRC tissues for β-catenin and IGF2. As shown in Fig. 6h, β-catenin and IGF2 protein levels in CRC tissues was higher than that in ANTs. As expected, we discovered a negative correlation between miR-486-5p and β-catenin as well as IGF2 (Fig. 6h). Additionally, in subcutaneous xenograft tumors from nude mice, PLAGL2, β-catenin, and IGF2 protein expression levels were lower in the LV-miR-486-5p group than that in the LV-NC group (Fig. 6i). Taken together, we concluded that miR-486-5p modulates CRC cell proliferation and migration via PLAGL2/β-catenin/IGF2 signaling pathways.

### Upregulated plasma miR-486-5p is tumor derived and acts as a potential diagnostic biomarker of CRC

**Fig. 7:**
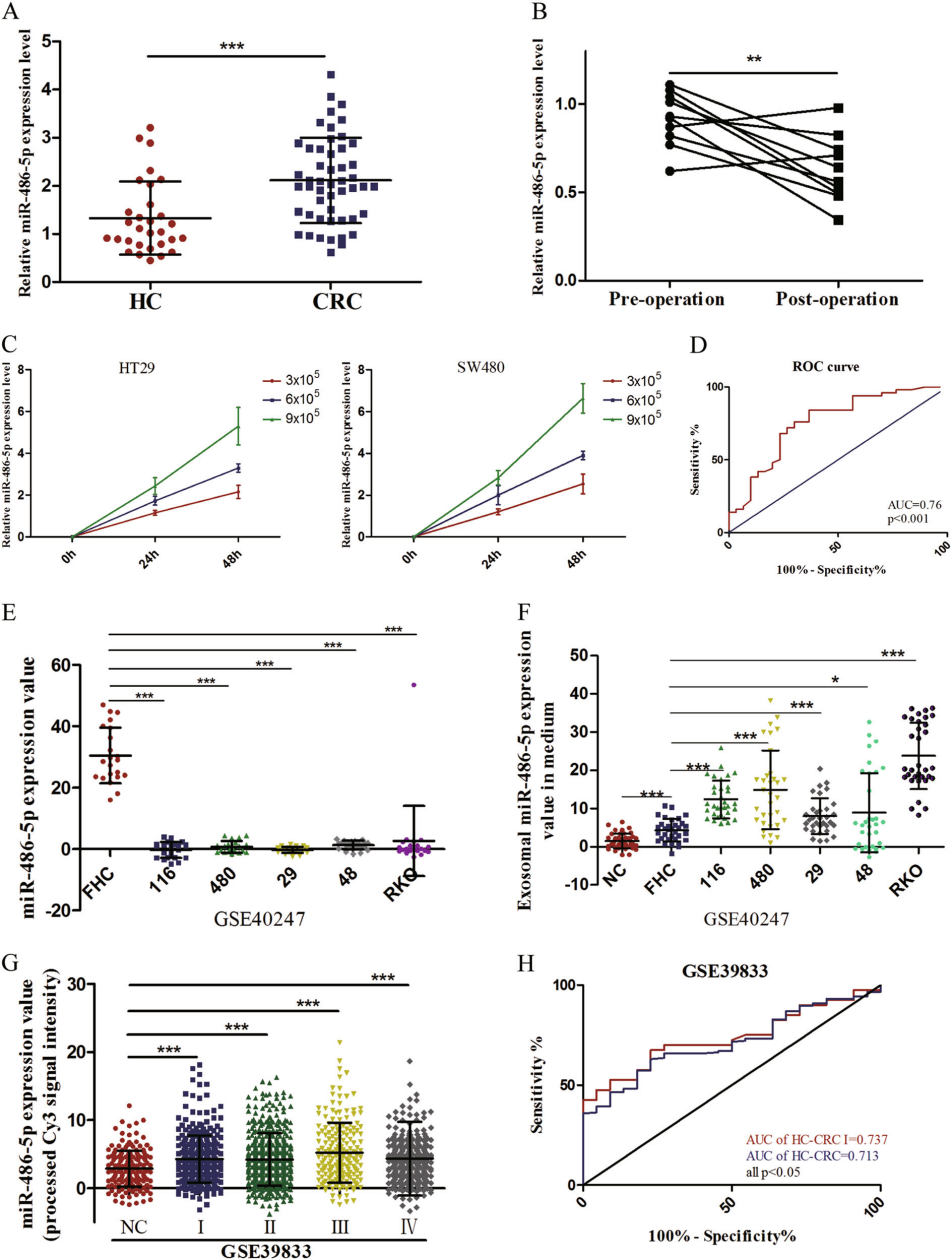
Upregulated plasma miR-486-5p is tumor derived and acts as a potential diagnostic biomarker of CRC. **a** Plasma miR-486-5p was significantly higher in CRC patients than that in healthy individuals. **b** MiR-486-5p were significantly downregulated after operation. **c** MiR-486-5p expression levels in the medium of HT29 and SW480 cells increased with increasing cell counts and incubation time. **d** The diagnostic utility of plasma miR-486-5p in CRC analyzed by ROC curve. **e** The expression of exosomal miR-486-5p was remarkably lower in CRC cell lines than that in FHC. **f** Exosomal miR-486-5p expression was significantly higher in the medium of CRC cells than that in the medium of FHC. **g** Circulating exosomal miR-486-5p expression was statistically upregulated in CRC patients with various stages. **h** The diagnostic utility of circulating exosomal miR-486-5p in CRC analyzed by ROC curve. Data are represented as means ± S.D. from at least three independent experiments. *p < 0.05, **p < 0.01, ***p < 0.001

Despite our results demonstrating that miR-486-5p is downregulated in CRC cell lines and tissues that is consistent with study of Liu et al.^25^, we discovered that plasma miR-486-5p was significantly higher in CRC patients than that in healthy individuals (Fig. 7a). To investigate whether plasma miR-486-5p was tumor derived, the expression of miR-486-5p were tested in 10 paired pre- and post-operative plasma samples. As shown in Fig. 7b, expression levels of plasma miR-486-5p were significantly lower after operation. In addition, we found that miR-486-5p expression levels in the medium of HT29 and SW480 cells increased with increasing cell counts and incubation time (Fig. 7c). These data revealed that miR-486-5p is a secretory miRNA. To explore the diagnostic utility of plasma miR-486-5p in CRC, receiver operating characteristic (ROC) curve analysis was carried out. The area under ROC curve (AUC) of miR-486-5p was 0.76 with 78% sensitivity and 62.3% specificity (Fig. 7d).

Several studies reported that some special RNA-binding proteins, such as hnRNPA2B1 and SYNCRIP, could control the sorting of endogenous miRNAs into exosomes through binding to specific motifs^26,27^. Therefore, we hypothesized that plasma miR-486-5p was secreted by CRC cells by being packaged into exosomes, which was proved by the results of GSE39833. As shown in Fig. 7e, the expression of miR-486-5p was remarkably lower in CRC cell lines than that in FHC. In contrast, exosomal miR-486-5p expression was significantly higher in the medium of CRC cells than that in the medium of FHC (Fig. 7f). Besides, circulating exosomal miR-486-5p expression was statistically upregulated in CRC patients with various stages (Fig. 7g). The AUC of miR-486-5p to discriminate CRC patients from healthy subjects was 0.713 with 67.5% sensitivity and 77.3% specificity and the AUC of miR-486-5p to distinguish CRC patients with stage I from healthy individuals was 0.737 with 63.1% sensitivity and 77.3% specificity (Fig. 7h).

## Discussion

**Fig. 8:**
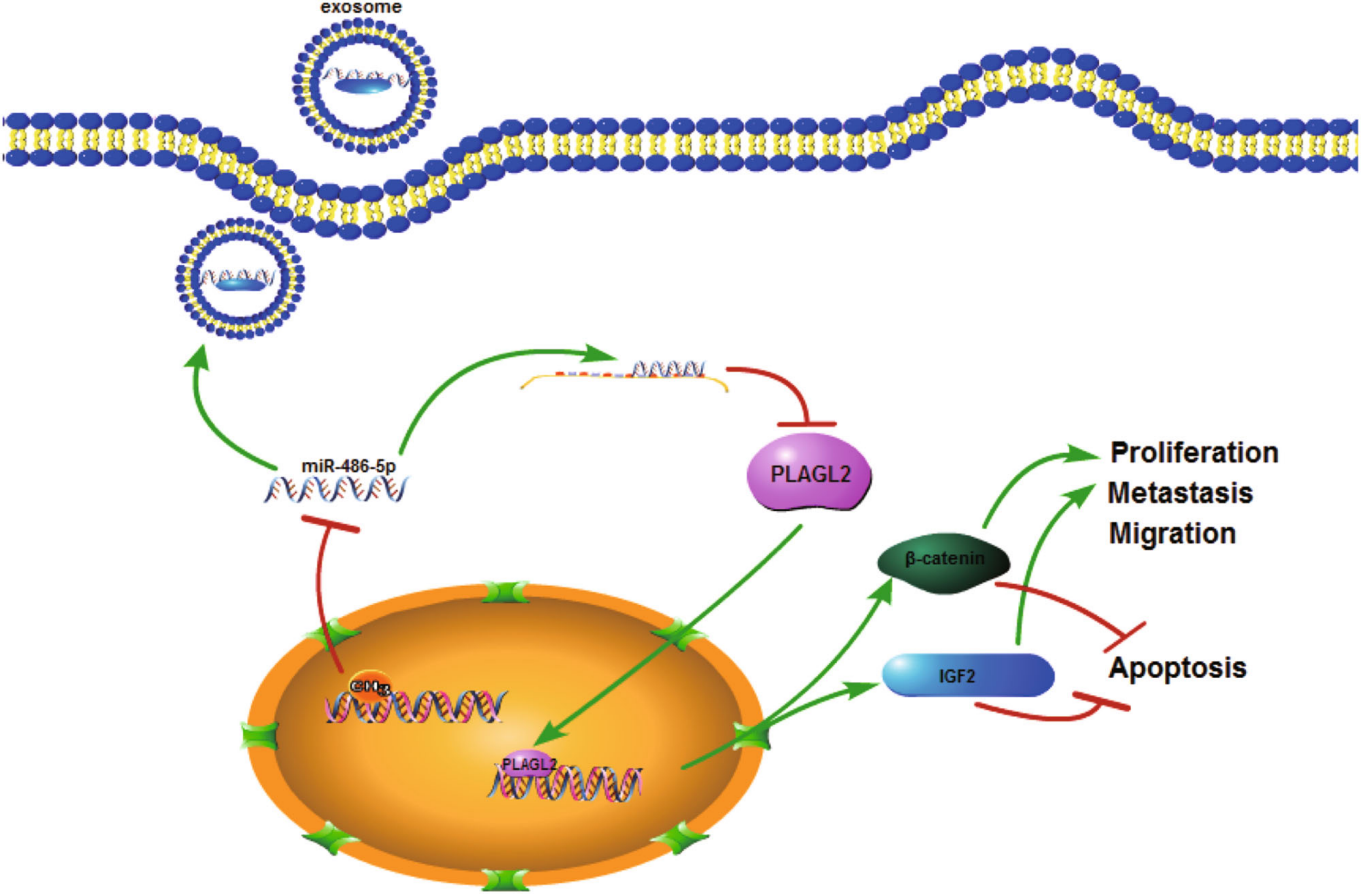
The schematic cartoon showing constitutive activation of the PLAGL2/IGF2/β-catenin signal pathways mediated by epigenetic silencing of miR-486-5p contributes to CRC development and progression

Emerging evidence has revealed that miR-486-5p acts as an oncogene or tumor suppressor in various cancers, including non-small cell lung cancer, prostate cancer, esophageal squamous cell carcinoma, and papillary thyroid carcinoma^12,28–30^. However, its biological role in CRC remains elusive. In this study, we demonstrated that miR-486-5p was downregulated in CRC partly owing to higher DNA methylation level of the promoter region, which contributed to poor prognosis of patients. In addition, miR-486-5p inhibited cell proliferation and migration of CRC cell lines through direct targeting PLAGL2, thus suppressing downstream genes' expression, such as IGF2 and β-catenin. Interestingly, we discovered that plasma miR-486-5p was tumor derived and upregulated in CRC and served as a potential diagnostic biomarker of CRC with non-invasion (Fig. 8).

Liu et al. declared that miR-486-5p acts as an oncogene in CRC and attenuates tumor growth and lymphangiogenesis by targeting neuropilin-2^25^. In spite of the previous study reporting that miR-486-5p was downregulated in CRC that is consistent with our results, we further validated its oncogenic role through exploring TCGA, GEO, and Starbase 2.0 databases. Recently, epigenetic regulators, such as transcription factor and DNA methylation, have been reported to modulate the expression of miRNAs^7,31,32^. Sabine et al. identified miR-486-5p as a direct target of the P53-induced miRNAs in CRC by chromatin-immunoprecipitation and sequencing analysis^33^. In addition to the regulation of transcription factor P53, in our current study, we are the first to reveal the downregulation of miR-486-5p, at least in part, due to its higher DNA methylation level in its promoter region.

Shino et al. reported that miR-486-5p was downregulated in CRC tissues and was a predictive biomarker for the efficacy of vaccine treatment in metastatic CRC^34^. Interestingly, in contrast to the low expression of miR-486-5p in CRC tissues, we found that plasma miR-486-5p expression was significantly upregulated in CRC patients. Yan et al. once suggested that exosome-encapsulated miR-486-5p in serum may serve as a disease biomarker for CRC by microarray analysis^35^. In this study, we identified plasma miR-486-5p as a novel potential biomarker for CRC diagnosis with non-invasion and found that plasma miR-486-5p was tumor derived. GEO dataset (GSE39833) further validated our guess that, despite the low miR-486-5p in CRC tissues, miR-486-5p could be packaged into secretary exosomes, thus resulting in upregulation of miR-486-5p in peripheral blood. Several RNA-binding proteins, such as hnRNPA2B1 and SYNCRIP, were reported to control the sorting of miRNAs into exosomes through binding to specific motifs^26,27^. Therefore, the specific RNA-binding protein that regulates the sorting of miR-486-5p into exosomes needs to be further investigated.

PLAGL2, a well-known transcription factor, was proposed to participate in the physiological regulation of different types of cells, including cancer cells^21,22,24,36^. Liu et al. reported that PLAGL2 expression was significantly higher in CRC tissues than in ANTs and correlated with the depth of tumor invasion^37^. Landrette et al. demonstrated that PLAGL2 is a novel leukemia oncogene that functions by inducing acute myeloid leukemia in cooperation with Cbfb-MYH11^18^. Another study illustrated that PLAGL2 expression is associated with the development of lung adenocarcinoma and emphysema^38^. However, the posttranscription regulation of PLAGL2 by miRNAs remains unclear. Using four different miRNAs target-predicting software, TCGA database, WB, and dual-luciferase reporting assay, PLAGL2 was proved to be a direct target gene of miR-486-5p. In our study, we demonstrated that PLAGL2 could promote cell proliferation and migration of CRC through inducing IGF2 and β-catenin expression.

In conclusion, our study provided robust evidence that miR-486-5p acted as a tumor-suppressor gene to inhibit CRC cells' proliferation and migration through regulating PLAGL2/IGF2/β-catenin expression and served as a novel prognostic and diagnostic biomarker of CRC. It is a promising therapeutic strategy of CRC treatment by targeting the new signal axis.

## Materials and methods

### Clinical samples

Fifty paired CRC tissues and ANTs were obtained from patients who underwent primary surgery resection at Nanjing First Hospital affiliated to Nanjing Medical University. All specimens were frozen in liquid nitrogen after surgery and pathologically confirmed until RNA extraction. Plasma specimens were collected from patients diagnosed as CRC or healthy people who had physical examinations. Plasma samples were stored at −80 °C until RNA extraction. None of the subjects recruited in this study received chemotherapy or radiation therapy before specimens were collected. Moreover, this study was approved by the ethics committee of Nanjing First Hospital, and written informed consents were obtained from all participants.

### GEO and TCGA database analysis

GEO datasets about miRNA expression in CRC were searched in GEO database using keywords “microRNA” and “colorectal cancer” and further analyzed by online tool GEO2R. The analysis of genes in TCGA database was conducted by R Studio or online tools LinkedOmics (http://www.linkedomics.org/admin.php) and UALCAN (http://ualcan.path.uab.edu/index.html).

### Cell cultures

The colonic mucosal epithelial cell (FHC) and CRC cell lines (HCT116, HCT8, SW480, SW620, and HT29) were purchased from American Type Culture Collection (Manassas, VA, USA) and had been tested and authenticated through STR (Short Tandem Repeat) method. Cells were subsequently cultured in Dulbecco’s Modified Eagle’s Medium (DMEM) supplemented with 100 µl fetal bovine serum (FBS) (Gibco, Vienna, Austria), 10 µl penicillin (Gibco, Vienna, Austria), and 10 µl streptomycin (Gibco, Vienna, Austria) per ml medium in humidified atmosphere containing 5% CO_2_ at 37 °C.

### RNA isolation, reverse transcription, and qRT-PCR

Total RNA was extracted from tissues, cells, plasma, and medium by using Trizol LS reagent (Invitrogen, CA, USA). The expression of miR-486-5p was determined by hairpin-it^TM^ microRNA Normalization RT-PCR Quantitation Kit (GenePharma, Shanghai, China). For mRNAs, RNA was first reverse transcribed to cDNA and then qRT-PCR was conducted using SYBR® Premix Ex TaqTM Kit (Takara, Otsu, Japan). GAPDH was used as the reference gene. U6 and cel-miR-39-3p (GenePharma, Shanghai, China) was chosen as the internal control and external reference for miRNA expression, respectively. The relative expression of miR-486-5p and mRNAs was calculated using 2^−^^ΔΔCt^ method. The primer sequences of mRNAs are listed in Table 3.

**Table 3:** Primer sequences of genes in the study

Name	Primer sequences
PLAGL2	Forward: 5′-AAAGCAGGAGGAGGAAGTGG-3′
	Reverse: 5′-TTTCTGGGCTGAGTGGGTGG-3′
ARID4B	Forward: 5′-GGCATAGGTTATTTCCGTGGTA-3′
	Reverse: 5′-CTTTGGCACATTTTTATCAGCA-3′
GAPDH	Forward: 5′-GAAGGTGAAGGTCGGAGTC-3′
	Reverse: 5′-GAAGATGGTGATGGGATTTC-3′
MSP (methylation)	Forward: 5′-CGTCGATGTCGTTTAGGAGTAC-3′
	Reverse: 5′-GCGAACGCCTCAATACCT-3′
MSP (unmethylation)	Forward: 5′-TAGTGTTGATGTTGTTTAGGAGTAT-3′
	Reverse: 5′-ACCACAAACACCTCAATACCT-3′
BSP	Forward: 5′-AGGGTTTTATTATGTTGGTTAGG-3′
	Reverse: 5′-CCTCAATACCTTAAAATATTTCATAAA-3′

### MSP, BSP, and DNA demethylation treatment

Genomic DNA was extracted from CRC and normal tissues by using the DNeasy Blood and Tissue Kit (Qiagen, Duesseldorf, Germany) and then exposed to bisulfite using an EZ DNA Methylation-Gold^TM^ Kit (Zymo Research, CA, USA) according to the manufacturers’ instructions. Bisulfite converted genomic DNA was chosen as template for MSP. Methylated as well as unmethylated control DNAs were used as a reaction control for MSP (Qiagen, Duesseldorf, Germany). For BSP, bisulfite converted genomic DNA was amplified using BSP primers and then cloned into pGEMT Easy vector (Promega, WI, USA). Thereafter, ten clones from each sample were sequenced using an ABI3130x automated sequencer (Applied Biosystems, CA, USA). Data were analyzed using a BIQ Analyzer (http://biq-analyzer.bioinf.mpi-inf.mpg.de/). MSP- and BSP-specific primers were designed using Methyl Primer Express v1.0. For DNA demethylation treatment, CRC cells were treated 5-aza-2-deoxycytidine (Sigma, CA, USA) for 72 h at 37 °C with medium replaced every day. The primer sequences of MSP and BSP are listed in Table 3.

### Cell transfection and vector construction

MiR-486-5p mimics (miR-mimic), miR-486-5p inhibitors (miR-inhibitor), and their corresponding control oligonucleotides (NC and anti-NC) were synthesized by GenePharma (Shanghai, China). SiPLAGL2, a small interfering RNA against PLAGL2, were synthesized by RiboBio (Guangzhou, China). Transfection was carried out with a final concentration of 50 nM miR-486-5p mimics and siPLAGL2 and 100 nM miR-486-5p inhibitors using the Lipofectamine 2000 reagent (Invitrogen, CA, USA) following the manufacturer’s protocol.

Lentiviral miR-486-5p (LV-miR-486-5p) along with lentiviral miR-NC (LV-NC) were purchased from Genechem (Shanghai, China), which carried a green fluorescent protein sequence and puromycin sequence. In order to select the stable cell lines, lentivirus-transfected HT29 cells were cultured in medium with 1 µg/ml puromycin for 14 days.

### Colony-formation assay

Twenty four hours after transfection, 500 uniparted HT29 and SW480 cells were planted into 6-well plates and cultured for 2 weeks. Thereafter, the cell colonies were fixed with methanol for 5 min and stained with 0.1% crystal violet for 15 min at room temperature. Then cell colonies were counted and photographed.

### EdU incorporation assay

The EdU assay was conducted using a Cell-Light EdU DNA Cell Proliferation Kit (RiboBio, Guangzhou, China). Briefly, 2 h after incubation with EdU, HT29 and SW480 cells were fixed with 4% paraformaldehyde followed staining with Apollo Dye Solution and then mounted with Hoechst 33342. The positive cells were photographed and counted using an Olympus FSX100 microscope (Olympus, Tokyo, Japan).

### Apoptosis analysis

Forty eight hours after transfection, 3 × 10^5^ cells were collected and washed with phosphate-buffered saline (PBS) twice. Cell apoptosis was analyzed with the Annexin V-fluorescein isothiocyanate/propidium iodide Apoptosis Detection Kit (BD Biosciences, CA, USA) and then analyzed by fluorescence-activated cell sorting using FACScan (BD Biosciences, CA, USA).

### Migration assay

After transfection, digested HT29 and SW480 cells were planted into 6-well plates and cultured for 24 h. Then 200 μl pipette tips were used to scratch three parallel lines and cells were washed with PBS twice, after which cells were cultured in an incubator at 37 °C. Photographs were taken at 0 and 24 h after wounding under Olympus FSX100 microscope (Olympus, Tokyo, Japan). Migration index was assessed by measuring the change of scratch area.

### Invasion assay

Matrigel mix (BD Biosciences, CA, USA) was used to coat the top chamber of transwell chambers (8 µm pore size) (BD Biosciences, CA, USA). Then 500 μl DMEM containing 10% FBS was added to the bottom chamber while 1.5 × 10^5^ cells were seeded in the top chamber. After 24 h, cells that had passed through the matrigel to the underside of the filter were fixed with methanol and stained by 0.1% crystal violet, otherwise these were removed using cotton swabs. Cells. Cells were counted under Olympus FSX100 microscope (Olympus, Tokyo, Japan) and the number of stained cells represented invasiveness.

### Dual-luciferase reporter assay

For the luciferase reporter assay, the wt and mut 3′-UTRs of PLAGL2 were amplified and inserted into pmirGLO luciferase vector (GeneCreat, Wuhan, China). The wt/mut vectors were co-transfected with miR-mimic or NC into 293T cells, respectively, using Lipofectamine 2000 in accordance with the procedure of manufacturer (Invitrogen, CA, USA). Forty eight hours after transfection, cells were harvested and luciferase activity was detected using the dual-luciferase reporter assay system (Promega, WI, USA). Renilla luciferase served as internal control.

### Western blotting

To harvest protein, cells were collected and lysed with RIPA lysis Buffer and then same amounts of denatured protein were separated by 10% sodium dodecyl sulfate-polyacrylamide gel electrophoresis followed by transfer to polyvinylidene difluoride membranes. The membranes were blocked with TBST containing 5% skimmed milk and subsequently blocked with primary antibodies overnight at 4 °C. After being washed, the membranes were incubated with horseradish peroxidase-conjugated secondary antibody for 1 h at room temperature. Bands were visualized using the enhanced chemiluminescence system reagent (KeyGEN BioTECH, Nanjing, China). The primary antibodies used were anti-PLAGL2 (ab121239, Abcam), anti-IGF2 (ab9574, Abcam), anti-β-catenin (ab32572, Abcam), anti-E-cadherin (20874-1-AP, Proteintech), anti-N-cadherin (22018-1-AP, Proteintech), anti-Vimentin (10366-1-AP, Proteintech), anti-ARID4B (24499-1-AP, Proteintech), and anti-β-actin (ab8227, Abcam).

### Immunohistochemistry

IHC for the target proteins was performed on formalin-fixed, paraffin-embedded tissue sections. The degree of positivity was initially classified according to scoring both the proportion of positive staining tumor cells and the staining intensities as previously described.^39^ For histological scoring, three high-power fields were randomly selected from CRC and normal tissues. The primary antibodies used were anti-PLAGL2 (ab121239, Abcam), anti-IGF2 (ab9574, Abcam), and anti-β-catenin (ab32572, Abcam).

### Animal experiments

For xenograft subcutaneous implantation model, 4-week-old male BALB/c nude mice were randomly divided into two groups (*n* = 5 for each group). After adapting to the new environment for a week, 5 × 10^6^ HT29 cells that had been stably transfected with LV-miR-486-5p or LV-NC were subcutaneously injected into the left oxter of nude mice. The tumor volume of each mouse was measured every week. Tumor volume = 1/2 (length × width^2^). Thirty days later, the nude mice were killed and the weight of tumors were recorded. Thereafter, the tumor tissues were further implemented hematoxylin and eosin (HE) staining and IHC staining.

For pulmonary metastasis model, 10 5-week-old male BALB/c nude mice were randomly divided into 2 groups, and 1 × 10^6^ stably transfected HT29 cells were injected into each nude mouse through tail vein. Seven weeks later, the mice were sacrificed and the lungs were surgically resected. The number of visible pulmonary metastasis nodules in each mouse was counted by two pathologists. Whereafter, the lung tissues were fixed in 4% paraformaldehyde, followed by HE staining. The animal experiments were approved by the Institutional Animal Care and Use Committee of Nanjing Medical University.

### Statistical analysis

All data are expressed as mean ± SD (standard deviation) from at least three independent experiments. Statistical analysis of the differences between groups were performed by SPSS 19.0 (IBM, USA) or GraphPad Prism 5.0 (GraphPad Software, USA) using the Student’s paired or unpaired *t* test or one-way analysis of variance. Kaplan–Meier method with the log-rank test was used to calculate OS rate. Cox proportional hazards model was used for the univariate and multivariate analysis. ROC was performed to evaluate the utility of plasma miR-486-5p as a diagnostic biomarker of CRC. *p* < 0.05 was considered to be statistically significant.
